# A Comprehensive Evaluation of Setup Uncertainties and Margin Optimization Using Deep Inspiration Breath-Hold Versus Free-Breathing Techniques in Breast Carcinoma

**DOI:** 10.7759/cureus.106419

**Published:** 2026-04-04

**Authors:** Jeeva Sivasamy, Indhumathi Jayaraman, Satheesh Kumar Anbazhagan, Vishnu Alagarsamy

**Affiliations:** 1 Radiation Oncology, Madras Medical College, Chennai, IND; 2 Medical Physics, Madras Medical College, Chennai, IND

**Keywords:** breast carcinoma, deep inspiration breath-hold, free breathing, planning target volume, setup uncertainties

## Abstract

Purpose: To systematically evaluate and compare geometric setup errors and calculate optimal planning target volume (PTV) margins for patients with left-sided breast carcinoma treated with three-dimensional conformal radiotherapy (3D-CRT) using free-breathing (FB) versus deep inspiration breath-hold (DIBH) techniques.

Materials and methods: A retrospective analysis was conducted on 103 postoperative breast carcinoma patients (n = 47 in the FB group; n = 56 in the DIBH group). Daily setup verification was performed using paired kilovoltage (kV) orthogonal images, yielding 348 analysed fractions. Setup deviations were measured in anterior-posterior (AP), superior-inferior (SI), and right-left (RL) directions. PTV margins were derived using the Van Herk formula.

Results: In the FB group, systematic errors were 0.16 cm (AP), 0.17 cm (SI), and 0.17 cm (RL), with random errors of 0.28 cm, 0.37 cm, and 0.29 cm, respectively. In the DIBH group, systematic errors were 0.14 cm (AP), 0.16 cm (SI), and 0.16 cm (RL), with random errors of 0.25 cm, 0.29 cm, and 0.27 cm, respectively. DIBH significantly reduced setup errors in the SI direction (p = 1.1 x 10^-5^). Calculated PTV margins for DIBH were smaller, particularly in the SI axis (0.61 cm vs. 0.69 cm).

Conclusion: DIBH significantly mitigates random setup errors in the longitudinal direction by stabilizing diaphragmatic motion, allowing for reduced PTV margins and improved sparing of organs at risk.

## Introduction

Breast cancer is the most frequently diagnosed malignancy in women globally, representing a significant proportion of all new cancer diagnoses worldwide [1]. Adjuvant radiotherapy significantly reduces locoregional recurrence but poses risks to organs at risk, particularly the heart and lungs in left-sided disease [2,3]. Deep inspiration breath-hold (DIBH) has emerged as a standard motion management strategy to displace the heart away from high-dose fields and minimize cardiac toxicity [4,5]. However, geometric uncertainties during treatment delivery necessitate an adequate planning target volume (PTV) margin to ensure target coverage [6]. This study aims to quantify geometric uncertainties in three-dimensional conformal radiotherapy (3D-CRT) for free-breathing (FB) and DIBH techniques, and to derive optimal PTV margins using the established Van Herk formula [7]. The primary endpoint of this study is the quantification of the reduction in systematic setup error, random setup error, and the derived PTV margins in each axis using DIBH.

## Materials and methods

Ethics

The procedures followed were in accordance with the ethical standards of the responsible institutional committee on human experimentation and with the Helsinki Declaration of 1975, as revised in 2013. Institutional Ethics Committee approval was obtained prior to the study. Informed consent was obtained from all adult research participants, and patient confidentiality was ensured by anonymizing all data.

Selection and description of participants

A total of 103 female patients with left-sided breast carcinoma treated with adjuvant 3D-CRT were selected for this retrospective single-institution study. Participants were stratified into two cohorts: the FB group (n = 47) and the DIBH group (n = 56). Patients in the DIBH group were selected based on their ability to maintain a breath-hold for > 20 seconds and left-sided breast cancer. All patients were immobilized in the supine position on a carbon fiber breast board with arms elevated above the head to minimize skin folds and allow tangential field access [8].

Technical information

Daily setup verification was performed using orthogonal kilovoltage (kV) planar imaging via the Varian On-Board Imager (OBI) system (Varian Medical Systems, Palo Alto, CA). Images were acquired in the AP and lateral directions and registered to digitally reconstructed radiographs (DRRs) from the planning computed tomography (CT) scan, utilizing a bony anatomy match (focusing on the chest wall and ribs). A total of 348 image pairs were analyzed (187 for FB, 161 for DIBH), averaging approximately 3.4 imaging fractions per patient. All analyzed translational shifts represent pre-correction online setup data, recorded in three axes: vertical (anterior-posterior, AP), longitudinal (superior-inferior, SI), and lateral (right-left, RL).

Statistics

Statistical analyses were performed using Statistical Product and Service Solutions (SPSS; IBM SPSS Statistics for Windows, Armonk, NY) software. For each patient and axis, the mean shift and standard deviation were calculated. Population statistics were derived as systematic error (Σ), defined as the standard deviation of individual mean setup errors, and random error (σ), defined as the root mean square of individual variances. PTV margins were calculated using the Van Herk formula to ensure 95% dose coverage to the clinical target volume for 90% of the population: PTV = 2.5∑ + 0.7σ. To account for the intra-patient correlation caused by repeated measures (multiple imaging fractions per patient), differences in variances between groups were compared using a linear mixed-effects model.

## Results

Patient characteristics for both the FB and DIBH cohorts are summarized in Table 1. The DIBH technique demonstrated a consistent reduction in both systematic and random errors compared to the FB cohort. Detailed pre-correction setup errors for both populations across the three translational axes are summarized in Table 2. While initial systematic errors (Σ) were relatively comparable between the two groups - ranging from 1.6 mm to 1.7 mm for FB and 1.4 mm to 1.6 mm for DIBH - the random errors (σ) saw more notable decreases with breath-holding. In the AP and RL axes, the random errors for DIBH were reduced to 2.5 mm and 2.7 mm, respectively.

**Table 1:** Patient characteristics *P-values calculated using a Mann-Whitney U test for continuous variables and a chi-square test for categorical variables. DIBH: Deep inspiration breath-hold

Characteristic	Free-breathing (n=47)	DIBH (n=56)	Total (n=103)	P-value*
Age (years)				
Median	58	54	56	0.45
Range	35-78	32-72	32-78	
Body Mass Index (kg/m^2^)				
Median	27.4	25.8	26.5	0.12
Range	19.5-38.2	18.5-35.4	18.5-38.2	
Tumor Stage				
Stage I	18 (38%)	24 (43%)	42 (41%)	0.78
Stage II	22 (47%)	25 (45%)	47 (46%)	
Stage III	7 (15%)	7 (12%)	14 (13%)	

**Table 2:** Population setup errors (mm) with 95% confidence intervals AP: Anterior-posterior; DIBH: Deep inspiration breath-hold; FB: Free-breathing; RL: Right-left; SI: Superior-inferior. CI: Confidence interval

Direction	Group	Mean shift (mm) (95% CI)	Systematic error (Σ) (mm) (95% CI)	Random error (σ) (mm) (95% CI)
AP	FB	0.59 (-0.15 to 1.33)	1.57 (1.31-1.97)	2.82 (2.41-3.23)
AP	DIBH	0.04 (-0.62 to 0.70)	1.45 (1.22-1.78)	2.52 (2.15-2.89)
SI	FB	1.11 (0.24 to 1.98)	1.75 (1.46-2.20)	3.66 (3.13-4.19)
SI	DIBH	-0.30 (-1.08 to 0.48)	1.65 (1.39-2.03)	2.87 (2.45-3.29)
RL	FB	-0.10 (-0.85 to 0.65)	1.75 (1.46-2.20)	2.93 (2.50-3.36)
RL	DIBH	-0.08 (-0.80 to 0.64)	1.60 (1.35-1.97)	2.68 (2.29-3.07)

The most pronounced geometric improvement was observed in the SI direction. The FB group exhibited its largest mean shift (1.11 mm) and highest random error (3.66 mm) along this longitudinal axis. In contrast, the implementation of DIBH suppressed this movement, yielding a smaller mean shift (-0.30 mm) and lowering the random error to 2.87 mm. Furthermore, threshold analysis revealed that 100% of the DIBH shifts in the SI direction were contained within a 7 mm boundary, which emerged as the minimum rounded whole-number value encompassing all observed deviations in this cohort. In contrast, only 98.9% of shifts in the FB group fell within this same data-derived threshold, further illustrating the superior geometric containment achieved with breath-holding.

Applying the Van Herk formalism (PTV = 2.5Σ + 0.7σ), the required PTV margins were consistently lower for the DIBH cohort across all evaluated directions (Table 3). The margin calculations for the AP and RL axes showed modest reductions of 0.5 mm each for the DIBH group. However, the most substantial and clinically relevant difference occurred in the SI axis, where the required margin dropped from 6.9 mm for FB patients to 6.1 mm for DIBH patients. This 0.8 mm margin reduction in the SI direction was driven by the aforementioned decrease in random error and was found to be highly statistically significant (p = 3.2 x 10^-4^).

**Table 3:** Calculated PTV margins and statistical significance *P-values calculated using a linear mixed-effects model to account for intra-patient correlation (repeated imaging fractions). AP: Anterior-posterior; DIBH: Deep inspiration breath-hold; FB: Free-breathing; RL: Right-left; SI: Superior-inferior

Direction	FB margin (mm)	DIBH margin (mm)	Reduction (mm)	P-value*
AP	5.9	5.4	0.5	0.092
SI	6.9	6.1	0.8	3.2 x 10^-4^
RL	6.4	5.9	0.5	0.865

## Discussion

The primary finding of this retrospective analysis of 348 imaging fractions is the statistically significant reduction in random setup errors along the longitudinal (SI) axis when utilizing the DIBH technique (p = 3.2 x 10^-4^). Consequently, the calculated PTV margins necessary to ensure adequate target coverage were reduced from 6.9 mm in the FB cohort to 6.1 mm for DIBH patients. Modest margin reductions were also noted in the AP and RL axes, demonstrating an overall improvement in geometric stability when breath-holding techniques are applied.

Interpretation and implications

These findings align strongly with the physiological mechanisms underlying DIBH [8,9]. By maximizing inspiration and holding their breath, the patient voluntarily stabilizes the diaphragm, which serves as the primary driver of longitudinal motion in the thoracic cavity [8,9]. In standard FB, the continuous tidal excursion of the diaphragm causes the chest wall and underlying structures to move predominantly in the SI direction [9]. This continuous intrafraction motion contributes heavily to the higher random errors (3.66 mm) observed in our FB cohort [10]. The immobilization of the diaphragm during DIBH effectively isolates and suppresses this motion component, dropping the random error to 2.87 mm [10].

The clinical and dosimetric implications of reducing the PTV margin, even by fractions of a millimeter, are conceptually important [11]. A tighter posterior margin allows for more aggressive shielding of the ipsilateral lung and the heart [5]. This potentially reduces the mean heart dose and the volume of the lung receiving high-dose radiation, incrementally mitigating the well-documented risks of late-onset ischemic heart disease and radiation pneumonitis [3,12]. While our study relied on daily orthogonal kV imaging for setup verification, the future integration of surface-guided radiation therapy (SGRT) could provide continuous intrafraction monitoring to potentially refine these PTV margins even further [13,14].

Targeted cardiac substructure sparing

Beyond generalized whole-heart dose reductions, the geometric stabilization afforded by DIBH is particularly critical for the left anterior descending (LAD) coronary artery [15]. The LAD is situated on the anterior surface of the heart, placing it in immediate proximity to the tangential radiation fields used in left-sided breast treatments. Even minor intrafractional variations in the SI or AP directions during FB can inadvertently draw the LAD into the high-dose region, exacerbating long-term cardiac toxicity risks [16]. Our observed reduction in random errors through breath-holding tightly controls the spatial gradient near these critical substructures, offering a modest geometric advantage that may incrementally contribute to the sparing of organs at risk [17]. Future dosimetric validation is required to quantify the true clinical magnitude and cardiac benefit of this specific margin reduction.

Furthermore, while the Van Herk margin formula provides a mathematically robust rationale for our observed margin reductions, its core assumptions must be carefully contextualized within a DIBH framework. The model inherently assumes a normal (Gaussian) distribution of independent random and systematic errors, an infinite number of treatment fractions, and rigid-body anatomical mechanics. However, the physiological reality of DIBH introduces complex variables that can challenge these population-based assumptions. Patients may exhibit systematic, non-Gaussian intrafraction drifts - such as progressive fatigue causing an under-shooting of the breath-hold amplitude over the course of treatment - as well as non-rigid chest wall and pulmonary deformations. Consequently, while applying this standard formalism supports the safety of reducing PTV margins in DIBH cohorts, clinicians must remain cognizant that a purely Gaussian-based formula may slightly underestimate the true dosimetric impact of these individualized, non-rigid respiratory dynamics.

Workflow feasibility and compliance

From a practical perspective, the implementation of DIBH does introduce slight increases in initial simulation and daily treatment times due to the necessity of coaching and monitoring breath-holds [18]. However, the literature indicates that the vast majority of breast cancer patients can comfortably tolerate the procedure and maintain the required breath-hold duration across a standard fractionation schedule [19]. The substantial geometric benefits observed in our data reinforce that these modest workflow adaptations are highly justified.

Strengths and limitations

A major strength of this study is the comprehensive analysis of a large dataset comprising over 300 orthogonal image pairs in a real-world, high-volume clinical setting, providing a highly representative sample of daily setup variations. However, the non-random allocation of patients to the DIBH cohort introduces potential selection bias. Because patients capable of sustained breath-holds were preferentially selected, they likely possessed better baseline performance status and respiratory capacity, representing a more compliant or physiologically stable subgroup. This inherent bias may affect the generalizability of the reported reductions in setup uncertainties. Another notable limitation is the study's reliance on planar kilovoltage (kV) imaging for daily setup verification, which inherently does not capture rotational or volumetric uncertainties. The absence of volumetric imaging, such as cone-beam computed tomography (CBCT), impacts the overall accuracy of the setup error estimation by failing to account for three-dimensional tissue deformation. Finally, while DIBH successfully reduced random errors associated with respiration, systematic errors remained relatively comparable between the two groups. This indicates that initial patient positioning uncertainties are likely inherent to the carbon fiber breast board and arm-board immobilization system itself, rather than the chosen breathing technique [20]. Furthermore, investigations utilizing continuous portal imaging have corroborated that, while intrafraction motion during DIBH is typically minimal, continuous monitoring during beam delivery remains a valuable tool to verify intra-breath-hold stability and detect any subtle baseline drifts that static imaging might miss [21].

## Conclusions

This study demonstrates that the implementation of DIBH significantly enhances geometric stability during left-sided breast carcinoma radiotherapy compared to conventional FB techniques. By effectively neutralizing diaphragmatic excursion, DIBH substantially mitigates random setup uncertainties, most notably along the longitudinal superior-inferior axis, where respiratory motion is historically the most problematic. While baseline systematic errors related to patient immobilization remain a factor requiring daily image guidance, the suppression of intrafraction motion through breath-holding is a clear geometric advantage.

Consequently, the routine use of DIBH allows for a safe, mathematically sound reduction in PTV margins. These tighter margins directly translate to a decreased volume of healthy tissue within the high-dose irradiation field, offering a modest geometric advantage that may incrementally contribute to the sparing of critical organs at risk, such as the heart and ipsilateral lung. Ultimately, integrating DIBH with rigorous image verification optimizes both accurate target coverage and long-term patient safety, solidifying its essential role in modern breast radiotherapy workflows.

## References

[1] Bray F, Laversanne M, Sung H, Ferlay J, Siegel RL, Soerjomataram I, Jemal A (2024). Global cancer statistics 2022: GLOBOCAN estimates of incidence and mortality worldwide for 36 cancers in 185 countries. CA Cancer J Clin.

[2] McGale P, Taylor C, Correa C (2014). Effect of radiotherapy after mastectomy and axillary surgery on 10-year recurrence and 20-year breast cancer mortality: meta-analysis of individual patient data for 8135 women in 22 randomised trials. Lancet.

[3] Darby SC, Ewertz M, McGale P (2013). Risk of ischemic heart disease in women after radiotherapy for breast cancer. N Engl J Med.

[4] Hayden AJ, Rains M, Tiver K (2012). Deep inspiration breath hold technique reduces heart dose from radiotherapy for left-sided breast cancer. J Med Imaging Radiat Oncol.

[5] Korreman SS, Pedersen AN, Nøttrup TJ, Specht L, Nyström H (2005). Breathing adapted radiotherapy for breast cancer: comparison of free breathing gating with the breath-hold technique. Radiother Oncol.

[6] Stroom JC, Heijmen BJ (2004). Geometrical uncertainties, radiotherapy planning margins, and the ICRU-62 report. Semin Radiat Oncol.

[7] Van Herk M, Remeijer P, Rasch C, Lebesque JV (2000). The probability of correct target dosage: dose-population histograms for deriving treatment margins in radiotherapy. Int J Radiat Oncol Biol Phys.

[8] Wang X, Pan T, Pinnix C (2012). Cardiac motion during deep-inspiration breath-hold: implications for breast cancer radiotherapy. Int J Radiat Oncol Biol Phys.

[9] George R, Keall PJ, Kini VR (2003). Quantifying the effect of intrafraction motion during breast IMRT planning and dose delivery. Med Phys.

[10] Bruzzaniti V, Abate A, Pinnarò P (2013). Dosimetric and clinical advantages of deep inspiration breath-hold (DIBH) during radiotherapy of breast cancer. J Exp Clin Cancer Res.

[11] Topolnjak R, van Vliet-Vroegindewij C, de Ruiter P, Remeijer P, Rasch C, Sonke JJ (2012). Image guided radiotherapy for breast cancer patients: surgical clips as surrogate for the breast tumor bed. Radiotherapy and Oncology.

[12] Bornstein BA, Cheng CW, Rhodes LM, Rashid H, Stomper PC, Siddon RL, Harris JR (1990). Can simulation measurements be used to predict the irradiated lung volume in the tangential fields in patients treated for breast cancer?. Int J Radiat Oncol Biol Phys.

[13] Laaksomaa M, Sarudis S, Rossi M, Kapanen M, Peltola S (2024). Setup margins based on the inter- and intrafractional setup error of left-sided breast cancer radiotherapy using deep inspiration breath-hold technique (DIBH) and surface guided radiotherapy (SGRT). J Appl Clin Med Phys.

[14] Rong Y, Walston S, Welliver MX (2014). Improving intra-fractional target position accuracy using a 3D surface surrogate for left breast irradiation using the respiratory-gated deep-inspiration breath-hold technique. J Appl Clin Med Phys.

[15] Lorenzen EL, Taylor CW, Maraldo M (2013). Inter-observer variation in delineation of the heart and left anterior descending coronary artery in radiotherapy for breast cancer: a multi-centre study from Denmark and the UK. Radiother Oncol.

[16] Taylor CW, Wang Z, Macaulay E, Jagsi R, Duane F, Darby SC (2015). Exposure of the heart in breast cancer radiation therapy: a systematic review of heart doses published during 2003 to 2013. Int J Radiat Oncol Biol Phys.

[17] Latty D, Stuart KE, Wang W, Ahern V (2015). Review of deep inspiration breath-hold techniques for the treatment of breast cancer. J Med Radiat Sci.

[18] Bartlett FR, Colgan RM, Carr K (2013). The UK HeartSpare Study: randomised evaluation of voluntary deep-inspiratory breath-hold in women undergoing breast radiotherapy. Radiother Oncol.

[19] Remouchamps VM, Vicini FA, Sharpe MB, Kestin LL, Martinez AA, Wong JW (2003). Significant reductions in heart and lung doses using deep inspiration breath hold with active breathing control and intensity-modulated radiation therapy for patients treated with locoregional breast irradiation. Int J Radiat Oncol Biol Phys.

[20] Mast M, Leong A, Korreman S, Leeg G, Probsti H, Schererj P, Tsang Y (2023). ESTRO-ACROP guideline for positioning, immobilisation and setup verification for local and loco-regional photon breast cancer irradiation. Radiother Oncol.

[21] Lutz CM, Poulsen PR, Fledelius W, Offersen BV, Thomsen MS (2016). Setup error and motion during deep inspiration breath-hold breast radiotherapy measured with continuous portal imaging. Acta Oncol.

